# ENCODE and its first impractical application

**DOI:** 10.1186/2041-2223-4-4

**Published:** 2013-01-17

**Authors:** Bruce Budowle

**Affiliations:** 1 Department of Forensic and Investigative Genetics, Institute of Applied Genetics, University of North Texas Health Science Center at Fort Worth, 3500 Camp Bowie Blvd, Fort Worth, TX, 76107, USA

## 

The C value paradox, as initially coined, was encountered in early eukaryotic genomic studies with the oddity that genome size was not necessarily correlated with organism complexity [1]. With the discovery of non-coding DNA in the 1970s, it became apparent that the size of the eukaryotic genome was not related to the number of genes contained within it. Indeed, only a small portion (approximately 2%) of the human genome carries coding genes [2-4], the rest being the so-called “junk DNA” [5]. The human genome project further elucidated the number of genes in our genomes - counting a paltry 20,000 to 25,000 genes [2-4]. With so few genes one might ask “how could such a complex organism as *Homo sapiens* pass on the necessary genetic blueprint to the next generation?” An equally enticing question could be “how could nature be so wasteful and commit so much junk DNA to the human genome?” The Encyclopedia of DNA Elements (ENCODE) project has shed some light on these two questions. There is not one paper to cite but greater than 30 studies [6] that were coordinated and published in concert describing the results of a multi-year consortium effort to catalogue the functional elements of human DNA. Hundreds of authors reported on analyses of thousands of data sets. A good summary of the work is captured in the ENCODE Project Consortium’s September 2012 publication titled “An integrated encyclopedia of DNA elements in the human genome” [7]. The ENCODE project identified a large number of functional elements, defined as sites that encoded a product or exhibited a biochemical signature in the human genome. The power of current DNA sequencing technologies made the Consortium project possible. The depth of analysis is impressive. In this one paper more than 1,600 data sets were analyzed for a multitude of elements including human protein-coding and non-coding RNAs, pseudogenes, RNA from different cell lines, binding locations of a number of DNA-binding proteins and RNA polymerase components, DNase I hypersensitive sites, locations for histone modifications, and DNA methylation. The most exciting finding and one that may begin to address the two questions posed above was that 80.4% of the genome has a biochemical function, that is, it is covered by or near at least one ENCODE-identified element. More precisely, a large portion of the human genome contains a regulatory event. The authors state that “95% of the genome lies within 8 kilobases (kb) of a DNA–protein interaction…, and 99% is within 1.7 kb of at least one of the biochemical events measured by ENCODE.” The outcome is that the noncoding junk DNA is far from being useless genome filler. Instead, seemingly inert DNA can influence functional genes. The nature of genetic and epigenetic control is quite complex and exquisite and today all that more appreciated. ENCODE is a public resource that will contribute substantially to the understanding of gene expression and mechanisms of disease and, hopefully, cures.

Surprisingly though, what might be the first application of ENCODE data is not directed toward improving human health through molecular biology. Bolstered by the newly found functional nature of a greater portion of the junk DNA, an appeal in a U.S. Court has been brought forward in part on a basis that information derived from typing the short tandem repeat (STR) markers used in forensic human identification worldwide violates an individual’s privacy [8]. The argument exploits ENCODE data to suggest that there is some noticeable predictive power hidden with the forensic STRs related to the health status of an individual. After all the Consortium publication suggests that “Many discovered candidate regulatory elements are physically associated with one another and with expressed genes, providing new insights into the mechanisms of gene regulation. The newly identified elements also show a statistical correspondence to sequence variants linked to human disease, and can thereby guide interpretation of this variation.” Such ENCODE information should be understood and the limitations should be appreciated; we are far from extracting predictive power and unlikely to do so with the forensically-relevant STRs. Even without knowing any causal relationship, as now might be intimated by some with the ENCODE project data, association studies between the forensic STR loci and disease genes generally have come up empty, providing little if any predictive power. One could argue that one STR, the TH01 locus, has been shown to have some effect on expression at the gene *Tyrosine Hydroxylase*[9] well before the onset of the ENCODE project. Still armed with such information, there is little predictive power regarding an individual’s health status by knowing the allelic repeat state of the TH01 locus. Such limited power is not surprising; next generation sequencing and genome wide association studies overwhelmingly find that most diseases are genetically complex and one marker provides little value in determining risk of disease or outcome from therapeutics.

It would be a shame that the phenomenal effort that brought forth ENCODE might be misused to attempt to breach the foundations of forensic DNA typing. ENCODE’s value is in laying a foundation of the intricate functionality of the human genome that someday may help improve the human condition. Certainly, claims of privacy violations via human identification by STR typing are unfounded and criticizing this powerful forensic tool, based on ENCODE data, does not improve the human condition.
